# Self-monitoring and personalized feedback based on the experiencing sampling method as a tool to boost depression treatment: a protocol of a pragmatic randomized controlled trial (ZELF-i)

**DOI:** 10.1186/s12888-018-1847-z

**Published:** 2018-09-03

**Authors:** Jojanneke A. Bastiaansen, Maaike Meurs, Renee Stelwagen, Lex Wunderink, Robert A. Schoevers, Marieke Wichers, Albertine J. Oldehinkel

**Affiliations:** 1 University of Groningen, University Medical Center Groningen, Department of Psychiatry, Interdisciplinary Center Psychopathology and Emotion Regulation, Groningen, The Netherlands; 2 Friesland Mental Health Care Services, Department of Education and Research, Leeuwarden, The Netherlands

**Keywords:** Depression, Intervention, Self-monitoring, Personalized medicine, Experience sampling, Randomized Controlled trial, E-health

## Abstract

**Background:**

Depression is a leading cause of disability worldwide. To reduce the societal burden and improve quality of life for individual patients, treatments for depression need to be optimized. There is a particular need for person-tailored interventions that reinforce self-management of patients. Systematic self-monitoring and personalized feedback through the Experience Sampling Method (ESM) could provide such a person-tailored, empowering intervention that enhances treatment outcomes. The primary aim of this study is to investigate the efficacy of self-monitoring and personalized feedback as an add-on tool in the treatment of depressive complaints in a natural setting.

**Methods:**

The ZELF-i study is a pragmatic multi-site randomized controlled trial (RCT). We aim to recruit 150 individuals with depressive symptoms aged between 18 and 65 years, who have an intake for outpatient basic or specialized treatment at a mental health care organization in the North of the Netherlands. After the intake, participants will be randomly allocated to one of three study arms: two experimental groups engaging in 28 days of systematic self-monitoring (5 times per day) and receiving weekly personalized feedback on positive affect and activities (“Do”-module) or on negative affect and thinking patterns (“Think”-module), and a control group receiving no additional intervention. Self-report inventories of depressive symptoms, psychosocial functioning and feelings of empowerment will be administered before and after the intervention period, and at follow-up measurements at 1, 2, 3 and 6 months. The patient-experienced utility of the intervention will be investigated by a combination of quantitative and qualitative research methods.

**Discussion:**

The present study is the first to examine the effects of add-on self-monitoring and personalized feedback on depressive complaints in clinical practice. It is also the first to evaluate two different ESM modules targeted at both of depression’s core symptoms. Lastly, it is the first study that uses a combination of qualitative and quantitative methods to evaluate the patient-experienced utility of ESM with personalized feedback as an intervention for depression. Results of the present study may improve treatment for depression, if the intervention is found to be effective.

**Trial registration:**

Dutch Trial Register, NTR5707, registered prospectively 1 February 2016.

**Electronic supplementary material:**

The online version of this article (10.1186/s12888-018-1847-z) contains supplementary material, which is available to authorized users.

## Background

According to the World Health Organization, the leading cause of disability worldwide is depression [1], a common mental disorder that is characterized by two core symptoms: depressed mood and decreased interest or pleasure in activities [2]. Optimizing treatments for depression will reduce the societal burden and improve quality of life for individual patients. There is a particular need for cost-effective, personalized interventions that support the mental health care sector and reinforce self-management of patients (e.g., [3, 4]). Systematic self-monitoring and personalized feedback on contextualized patterns of affect through the Experience Sampling Method (ESM) could provide such an empowering, low-cost intervention.

With ESM, a patient gathers a multitude of prospective in-the-moment daily life assessments on affect, behavior, and context [5, 6]. Through aggregation of these systematically collected data, ESM can generate information that goes beyond what has been explicitly listed by the patient. Moreover, sophisticated analyses such as time-series analyses can determine the temporality of effects within a specific person, for instance, whether increased positive affect (PA) precedes or follows an increase in physical activity. ESM has generated important scientific insights in daily life emotional dynamics in depressed patients (for reviews see [7, 8]). Even more importantly, ESM also has great potential for clinical practice and the individual patient, because it allows personalized feedback (e.g., [9–11]. Small-scale proof-of-principle studies have underlined the potential of this approach for the individual patient (e.g., [10, 12, 13]). Kauer and colleagues [14] investigated the efficacy of self-monitoring and feedback on mood, stress, and daily activities as a therapeutic tool for adolescents with emotional or mental health issues in a larger study (*n* = 114). They found that, compared to the control group (which only monitored daily activities), emotional self-awareness increased more in the intervention group, which in turn decreased depressive symptoms. More recently, a pioneer randomized controlled trial (RCT) established the efficacy of ESM as a therapeutic tool for patients with depression (*n* = 102, [15]). That is, add-on ESM-derived personalized feedback on PA and activities resulted in a significantly and clinically relevant stronger decrease in depressive symptoms compared to pharmacological treatment alone. While promising, the ESM intervention was evaluated in the absence of psychotherapy, which is a common part of depression treatment, and had an extensive face-to-face component itself. Therefore, it is not yet known whether ESM as a self-management tool could have added positive effects to treatments in regular clinical practice. In addition, data acquisition as well as data analysis for the personalized feedback reports were a laborious exercise, hampering use in clinical practice.

We will take the necessary steps to move ESM-derived personalized feedback closer towards implementation as a therapeutic tool for depression in this study, which is named the ZELF-i project to emphasize the self-management aspect of our approach (self = *zelf* in Dutch, *i* stands for intervention). First, we will optimize the ESM intervention for clinical practice by making it easily accessible on patients’ own smartphones and by reducing personnel investment through automatized personalized analyses and digital feedback reports. Second, we will reexamine the previously reported efficacy of ESM-derived personalized feedback on PA and activities (ESM “Do-module”) in an RCT embedded in a naturalistic clinical context. Third, we will examine the efficacy of an alternative ESM module focused on negative affect (NA) and thinking patterns (ESM “Think-module”). Until now, there have only been studies with one specific module or a general set of items. Therefore, it is unknown whether the form (systematic self-monitoring) or the content of an ESM module is relevant. Our two ESM modules are both conceivably beneficial, as they link up with the two main angles of psychotherapy for depression: behavioral activation through positive reinforcement of activities, and cognitive therapy aimed at helping individuals recognize and replace negative thinking patterns [16].

Self-monitoring and personalized feedback through ESM is a potential improvement for depressed patients for several reasons. First, previous studies suggest clear health benefits in terms of a decrease in depressive symptoms [14, 15]. Second, in contrast to pharmacotherapy, the intervention mobilizes patients as active agents in their process to recovery, as evidenced by increases in feelings of empowerment [17]. Third, the intervention supports patients outside the clinician’s office, in their daily lives, which is where changes should occur. Fourth, person-tailored approaches should become the new focus in mental health care according to national and international directives (e.g., [4]) and patient organizations (e.g., *Landelijk Platform GGZ*). The intervention meets this need by providing personalized feedback on relevant aspects of daily life such as diurnal mood fluctuations, affective reactivity to social and physical activities, and the activation of negative thinking patterns in response to sad mood. Fifth, patients can start directly after intake, making the most out of the commonly long wait list period at mental health organizations [18]. Considering that patients usually start seeking professional care out of a sense of urgency, we hope that it will be satisfactory to them that they will be able to start working on their problems directly, without a period of passive waiting. Sixth, by bringing their feedback reports in at the start of the treatment, patients could commence specialized mental health care programs with a kick-start. By already having monitored their thoughts and feelings in relation to their activities for a couple of weeks, they are likely to have acquired a better insight in the processes involved in their depressive symptoms and day-to-day functioning than patients who did not partake in self-monitoring. Seventh, no costly equipment is required, only a working smartphone, which means the intervention could potentially reach many patients seeking care. In fact, interest among psychiatric outpatients in using smartphones to monitor their mental health is high [19].

According to guidelines for the development of evidence-based directives, two independently performed high-quality RCTs of substantive size provide the highest level of proof for the efficacy of an intervention [20]. Thus, the proposed RCT could provide more definitive answers regarding the efficacy of ESM as a therapeutic tool for depression. While a reduction in depressive symptomatology is important, we believe ‘health’ goes beyond the traditional definition as the absence of illness. ESM-derived personalized feedback could assist patients in actively trying to gain more control over their own psychological problems and enhance their functioning as evidenced by increases in feelings of empowerment [17] and increased engagement in social activities [21]. These functional outcomes are at least as relevant as clinical outcomes, as for many patients the essence of recovery is to rise above the presumed limitations associated with mental illness [22]. In fact, more and more voices are calling for a reformulation of ‘health’ into the ability to adapt and to self-manage in the face of social, physical and emotional challenges (e.g., [23]). Therefore, we will examine the impact of the ESM intervention not only on depressive symptoms, but also on measures of functioning and empowerment.

When evaluating an intervention not only the efficacy on a group level is important, but the perspective of individual patients also needs to be taken into account [24]. Qualitative methods in which the experiences and opinions of patients are at the center, should be an essential component of health care research [25]. Qualitative research based on interviews with patients has shown that an active attitude towards rehabilitation and structured attention to oneself are important themes for individuals with depression [26, 27]. ESM with personalized feedback could meet these wishes, but there has been virtually no research on patient experiences with such an intervention, except for a personal case study by Peter Groot [10]. He describes how self-monitoring of his mood helped him in his recovery process as follows (p. 354): *“I now look at my own mood swings in a different way. I can accept more easily the gloomy periods I still have once in a while; they are briefer, I get through them more easily, and I think they occur less frequently than in the past. I feel better ‘protected’ and more ‘empowered’ against the vulnerability with which I have apparently been cursed.”* It is not known whether these experiences generalize to other individuals. Therefore, a secondary aim of the ZELF-i project is to evaluate the patient-experienced utility of ESM with personalized feedback through patient interviews.

## Methods

ZELF-i is a multi-site trial designed according to the principles of a pragmatic RCT to allow evaluation of the intervention in real-life care facilities. Details are described below according to the SPIRIT guidelines [28]. The study was approved by the Medical Ethical Committee of the University Medical Center Groningen (no. 2015/530). The trial has been registered prospectively in the Dutch Trial Register (Nederlands Trial Register, NTR5707, http://www.trialregister.nl) at 1 February 2016.

### Participants

We will recruit 150 male and female adult patients referred for depressive complaints to general or specialized outpatient teams at mental health care organizations in the North of the Netherlands. Participants will be recruited within the research network of mental health institutions in the North of The Netherlands, the Rob Giel Research Center (https://www.rgoc.nl), which covers a catchment area of over 4 million inhabitants. Eligibility criteria are broad to include typical patients. Inclusion criteria are: a) indication for depression treatment by the mental health care professional (hereafter named: practitioner); b) age between 18 and 65 years; and c) written informed consent. Exclusion criteria are (based on practitioners’ appraisals): a) crisis intervention warranted (i.e., in the case of acute suicidality); b) presence of psychotic or manic symptoms; and c) incapability of following research procedures due to inadequate Dutch language proficiency, significant auditory or visual impairments or mental retardation. Patients will be enrolled in regular treatment upon availability, that is, the start of regular treatment will not be postponed until participation in ZELF-i finishes. The ZELF-i intervention is intended as an add-on tool at the start of care as usual and thus circumstances are otherwise kept as ‘natural’ as possible. Participants will receive travel reimbursements and an additional €10 if they used the data bundle of their own smartphone to participate in the intervention.

### Procedure

#### Recruitment

**Fig. 1:**
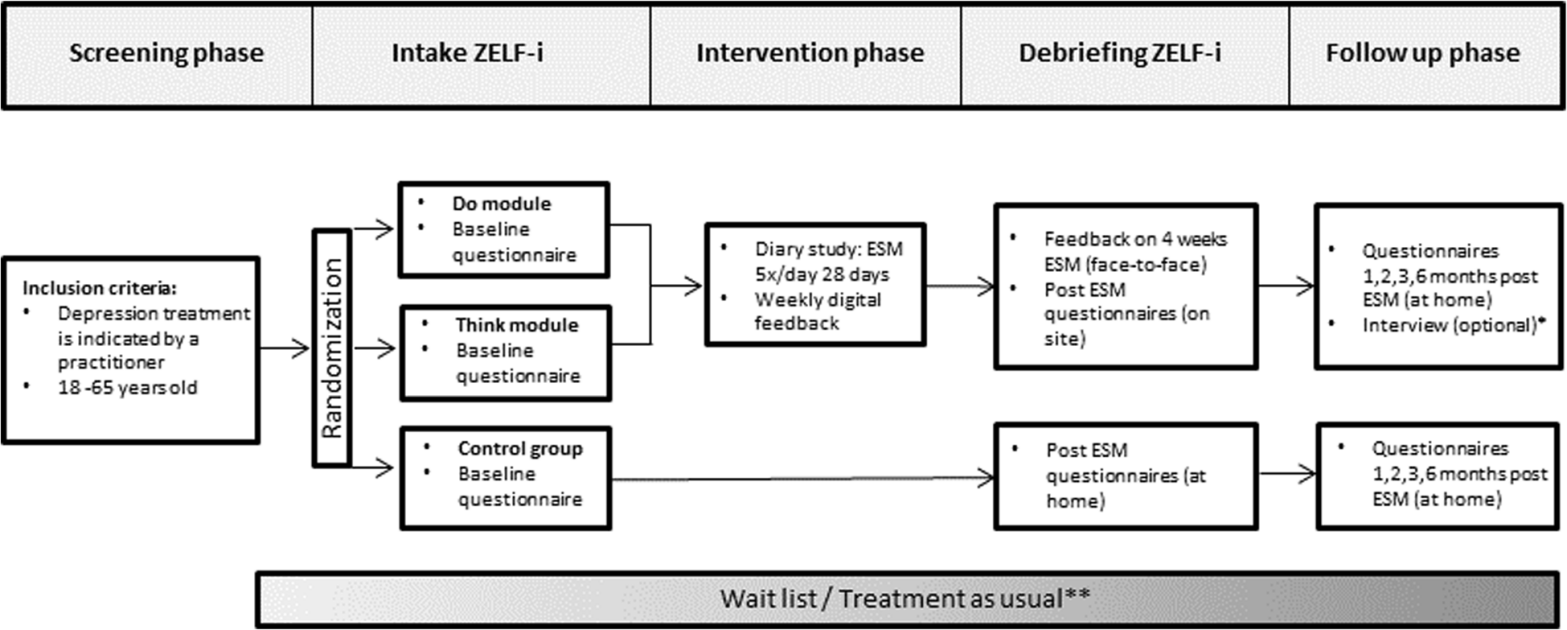
Overview of the design of the randomized controlled trial. ESM = Experience Sampling Method. * The interview is optional and will take place before the 2 month follow-up. ** The time interval between the start date of the participation in ZELF-i and the start date of regular treatment varies between participants

An overview of the study design is presented in Fig. 1. Individuals scheduled for intake at one of the participating mental health care teams will receive an information letter about ZELF-i and a contact consent form together with their intake invitation. Through the information letter, participants will be made aware of the procedure that, at the end of the intake, the practitioner will discuss the possibility to participate in ZELF-i with them if a) depression treatment is indicated, and b) the practitioner deems ZELF-i suited for the patient. Participants will be made aware that participation is voluntary and refusal to participate will not affect their treatment. Patients decide independently whether they are interested in the research and want to be contacted by the research team by sending back the consent form or bringing it to the intake at the mental health care institution. It is important that eligible participants are enrolled in the study shortly after the intake, because the intervention (28 days) is designed to support participants early on and bridge potential waiting periods between the intake and the start of the indicated depression treatment in a constructive way. Therefore, eligible participants will be contacted by a research assistant preferably within a few days after the intake to answer any questions and schedule an appointment for the introduction session of the intervention study.

#### Introduction session and baseline measurement

The introduction session will take place at the mental health care organization. At the start of the introduction session, participants will have another opportunity to ask questions about the study procedures. If everything is clear, they will be asked to provide written informed consent. After the informed consent procedure, participants will be randomly allocated to the control group or one of two ESM intervention groups. Then, detailed instructions and practice runs for the ESM modules follow, depending on the allocated treatment condition. Finally, all participants will fill out questionnaires assessing characteristics at baseline, depressive symptomatology, functioning, empowerment, and questionnaires necessary for cost-effectiveness analyses (see Study Parameters section).

#### Randomization

Participants will be randomly allocated to the control group or one of two ESM intervention groups (“Do-module” and “Think-module”). Randomization (allocation ratio 1:1:1) will be stratified per treatment location according to the duration of antidepressant pharmacotherapy (new/switch vs. maintenance, i.e., receiving antidepressant or mood stabilizing medication for less vs. longer than 8 weeks prior to study entry), and current psychotherapy (yes or no). For these different strata, separate blocks with an allocation sequence of six with an equal condition distribution will be created using an online randomization tool (https://www.random.org/lists). Thus, each of the three conditions will be present twice in a block of six. The allocation will be implemented by using sequentially numbered sealed envelopes (i.e., one pile of envelopes for each stratum). The allocation will be done during the introduction session, by team members who do not have access to the allocation sequence. Participants and research assistants are obviously not blind to treatment. Practitioners are not blind to treatment per se; participants decide themselves whether they want to discuss their feedback reports with their (future) therapist.

#### Intervention period

**Fig. 2:**
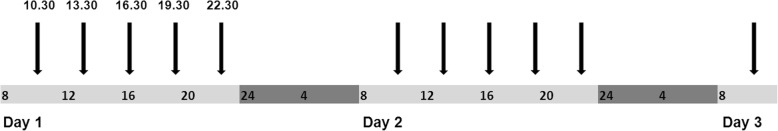
Example of sampling times for the self-report assessments. The total sampling period covers 28 days

The ESM measurements consist of brief questionnaires on the smartphone via a link to a secured website for routine outcome monitoring (RoQua, https://www.roqua.nl). Participants who do not have a smartphone of their own will be provided with one for use during the intervention period. Participants will be instructed how to fill out the ESM measurements using their smartphone and how to interpret the questions. Participants will fill out ESM measurements five times a day during the course of their daily lives for 28 days. The measurements will occur at set points in time during waking hours (i.e., every three hours during the day, Fig. 2), which will be programmed to optimally fit the participant’s usual daily rhythm.

Participants will be asked to complete their reports preferably immediately after they receive a text message with the link to the questionnaires. To minimize memory distortion, the link expires after 30 min. Participants will be sent a reminder text message after 15 min if they did not complete the measurement yet. During the ESM period, the registration of the questionnaires is regularly checked in RoQua. In case of > 10 subsequent missing measurements, participants will receive a telephone call to ask whether they want to stop the ESM measurements. Technical support will be available throughout the study.

For both ESM modules, each measurement starts with questions about the participants’ current mood (PA, NA) and physical state, followed by questions regarding activities for the Do-module and hassles/uplifts and positive/negative thoughts for the Think-module. In addition, the morning measurements include a question about sleep, and the evening measurements a few general questions on how participants experienced the past day and how they feel about the next day. The self-assessments will be rated on dichotomous scales (e.g., for the presence of hassles/uplifts) and visual analogue scales (usually ranging from not at all (0) to very much (100)). The items for the momentary questionnaires are presented in Additional file 1: Table S1.

Participants in the intervention groups receive personalized feedback after each intervention week based on the ESM measurements. This sums up to a total of 4 feedback moments. After weeks 1–3, feedback will be e-mailed to the participant in a digital report comprising text and simple graphs (e.g., pie charts, bar graphs). After week 4, a final feedback report will be e-mailed, in which participants will also receive feedback on temporal relationships between sets of variables (e.g., PA and physical activity (Do-module), or NA and rumination (Think-module)). To this end, time-series analysis will be employed on the individual time series at the end of the ESM period. Specifically, vector autoregression (VAR) models will be applied, which are particularly suited for the investigation of the temporal dynamics between two variables [29, 30].

For both ESM modules, the feedback reports will contain increasingly rich information across the intervention period by showing participants’ changes in affective responses in daily life situations from week to week. This way, the participants could become more aware of small improvements and get increased control over influences on their mood. In the Do-module, the feedback will also show changes in activities since the start of the study and ultimately provide information on the relationship between affective responses in daily life and activities in order to refocus and redirect the participants towards occasions for emotional strength. In the Think-module, the feedback will not only show participants’ changes in mood, but also negative and positive thinking patterns across the intervention period. The final report of the Think-module will provide information on the relationship between affective responses in daily life and thinking patterns such as rumination in order to make participants aware of potential negative spirals.

A research assistant will discuss the final feedback report with the participant during a debriefing session, which takes place at the mental health care organization shortly after the end of the intervention period. The research assistant will answer questions and help participants provide meaning to the graphs according to a standardized protocol. Personal notes will be made on the printed feedback report, which the participant will take home. Feedback reports are not sent to the (future) therapist by the researcher; participants can choose for themselves whether or not they want to share this information. After discussing the feedback report, participants fill out an evaluation questionnaire on the ESM intervention and the regular questionnaires (control participants fill out the latter at home). At a later point, the intervention tool will also be evaluated through an additional semi-structured interview in a subset of participants. Participants are provided with an information letter about this interview at the end of the debriefing session.

#### Follow up measurements

Consistent with a previous comparable RCT [15], participants will be invited (via e-mail) to fill-out questionnaires at 1, 2, 3 and 6 months post-ESM (online via the RoQua system). The participants will be able to fill out these forms at home. In case of non-response, participants will receive an e-mail and text message after 3 days to motivate them to fill out the questionnaires. A complete overview of the (timing of the) questionnaires is presented in Additional file 2: Table S2 in the Study Parameters section.

#### Interview

To get a better view on whether ZELF-i has added value to standard care, two psychologists will individually interview a subset of participants in the two intervention groups. The interviews will last for one hour and take place at the mental health care organization. Not all participants will be contacted for an interview; participants will be selected at random until data saturation is complete. Selected participants will be contacted by one of two psychologists by telephone within 8 weeks after the debriefing to answer questions and invite them to take part in the interview. Participation in the interview is optional, and independent of continued participation in the follow-up measurements. At the interview appointment, participants will be asked to sign a consent form specific to the interview. The interview will be recorded with audio equipment and processed into a transcript. The anonymized transcripts will be coded independently by two psychologist researchers, followed by a consensus discussion under supervision of a qualitative research expert. After data coding, the audio recordings will be destroyed.

### Data management

Data will be handled in a strictly confidential manner, complying with the Dutch Personal Data Protection Act (*Wet Bescherming Persoonsgegevens*). Each participant will be assigned a unique identification number in order to protect privacy. During data collection, team members will have access to the contact details of the participants in order to contact them. After data collection, only the principal investigator (JAB) of this study will have access to the coding of the subjects. Other investigators only have access to the coded files that do not contain information that can be traced to a specific individual. ESM and questionnaire data will be stored in Medoq, a research environment within RoQua, which cannot be accessed by practitioners. Participants choose themselves whether they want to share their feedback reports based on the ESM measurements with their practitioners. Any non-digitalized questionnaire data will be kept in locked cabinets in the investigator’s office.

As this intervention study does not include medical procedures we do not expect any serious adverse events to occur. Any undesirable experience reported spontaneously by the participants or observed by the investigator or her staff during the study, whether or not considered related to the experimental treatment, will be recorded as an adverse event. For each participating centre, the principle investigator will notify the coordinating investigator of any adverse and serious adverse events. A data monitoring committee has not been installed as the added risk in comparison to care as usual is negligible.

### Study parameters

The main endpoints are based on questionnaires, for which a complete overview is presented in Additional file 2: Table S2.

#### Main study parameter

The primary outcome measure to determine efficacy of the intervention will be the change in depression symptom severity as measured by the self-report Inventory of Depressive Symptomatology (IDS-SR, [31]) across 6 time points: baseline, after 4 weeks of ESM and at 4 follow-up measurements at 1, 2, 3, and 6 months (post-ESM). Participants will be followed prospectively to compare the efficacy of the intervention modules mutually and to the control group.

The 30-item self-report **Inventory of Depressive Symptomatology (IDS-SR)** includes all DSM-IV [2] diagnostic criterion items for major depressive disorder, as well as commonly associated symptoms such as irritability [31]. Each symptom item is scored on a scale from 0 to 3, with higher scores denoting greater symptom severity. The time frame for assessing symptom severity is the 7 day period prior to assessment (independent of whether symptoms have been long-standing, chronic, or recent). The IDS-SR is scored by summing the item responses. Either appetite increase or decrease, and either weight increase or decrease are used to calculate the score. If increase and decrease items are both completed, the highest of the two scores is used. The IDS-SR has good psychometric properties with high concurrent and internal validity (Cronbach’s α = .92) and is sensitive to treatment change [31, 32].

#### Secondary study parameters

From a patient perspective, functional outcomes are at least as relevant as clinical outcomes. Therefore, we will also assess change in psychosocial functioning by means of the Outcome Questionnaire (OQ-45) [33]. In addition, we will assess the extent to which individuals regain self-esteem and take control over their own lives by means of the Dutch Empowerment questionnaire (NEL) [34].

The **Outcome Questionnaire-45 (OQ-45)** is a 45-item self-report scale that measures subjective discomfort, disturbance in interpersonal relations, and functioning in social roles such as work and school [33]. Each item is scored on a 5-point scale from never (0) to almost always (4), yielding a range of possible scores of 0 to 180 with higher values indicating the endorsement of pathology. The OQ-45 can be used on a weekly basis and extensive validation in the United States has provided cut-off scores for reliable change and recovery as markers for gauging treatment success. Internal consistency (.90), test re-test reliability (.84 over 3 weeks in untreated participants), and concurrent validity with other scales are high [35]. The OQ-45 can be used regardless of the type of disorder or type of therapy. The Dutch version of the OQ-45 showed similar psychometric properties as the original instrument.

The **Dutch Empowerment questionnaire (NEL)** is a 40-item self-rating scale to assess patient empowerment, developed by the Dutch Trimbos Institute in collaboration with patients [34]. It incorporates six dimensions: professional help, social support, own wisdom, sense of belonging, self-management, and community inclusion. Items are formulated in positive statements of strengths as perceived by the individual and are rated on 5-point scales ranging from 1 (‘strongly disagree’) to 5 (‘strongly agree’), with the 4 items regarding professional health care having the additional option ‘not applicable’. The total score ranges from 40 to 200, with higher scores indicating more empowerment. Previous research has shown construct validity is satisfactory and internal consistency (α = 0.93) is high [34]. The latter has been corroborated by the ESM RCT in depression [17].

#### Other study parameters

To investigate whether ESM-derived feedback may be a cost-effective strategy in participants with depression from a societal perspective, we will administer questionnaires for costs due to illness and illness related costs (TiC-P: Trimbos/iMTA questionnaire for Costs associated with Psychiatric Illness [36], adjusted for a mental health population), and quality of life (Euroqol-5D) [37]. Similar questionnaires have also been used in the previous RCT [15] to perform cost-effectiveness and cost-utility analyses [17, 38]. In addition, we will extract information on care use in the 2-year period after participation in ZELF-i from the patient registries of the involved mental health care organizations and from the regional psychiatric case registry (https://www.rgoc.nl/#home/onderzoek/Register), which will be linked to our sample when approved by the participant (as stated on the informed consent form).

To assess patient-experienced utility of the ESM modules, we will administer an evaluation questionnaire after the ESM period. A small, randomly selected number of participants (see sample size calculation) will be invited to partake in a semi-structured interview with an experienced practitioner. Central topics in this semi-structured interview will include personal experiences with the intervention in relation to depressive complaints (burden, motivation), daily functioning, impact on daily rhythm, self-management, and self-insight. In addition, participants will be asked what improvements can be made in the ESM modules and how the intervention could be implemented in standard care. The interviews will generate qualitative data.

At baseline, sociodemographic characteristics (gender, age, educational level, work- and living situation), medication use, and current psychotherapy will be assessed by means of a questionnaire. Two additional questionnaires that will be administered are the Twenty-Item Toronto Alexithymia Scale (TAS-20) [39, 40] and the revised version of the Leiden Index of Depression Sensitivity (LEIDS-R) [41, 42]. Alexithymia (literally: “no words for emotions”) is a personality construct that is characterized by difficulties in identifying and describing emotions. Alexithymia is prevalent in approximately 10% of the general population, and 32%–51% of participants with depressive disorders [43, 44]. Since our intervention requires participants to repeatedly rate themselves on various emotional states, participants with high alexithymia scores may find it more difficult to benefit from the intervention. The LEIDS-R will be administered to assess cognitive reactivity, that is the degree to which negative thinking patterns are triggered by depressed mood. The intervention requires participants to focus on their mood states, and will possibly be less beneficial for participants who are highly cognitively reactive, because an increased focus on their mood may trigger negative thinking patterns.

### Sample size calculation

The RCT comprises nomothetic (between-person) and idiographic (within-person) aspects. The nomothetic aspect of the study is captured by questionnaire data (e.g. self-reports on depressive symptoms), which will be used for group-based analyses (e.g. tests of efficacy). The idiographic aspect is captured by the ESM measurements, which are used to provide participants with personalized feedback. These two different types of quantitative analyses require different sample size calculations.

#### Group analyses

In the previous RCT, IDS-SR scores for the experimental group (ESM + feedback) showed an initial 3-point drop 8 weeks after baseline, which increased to a 10-point drop compared to baseline at 32 weeks [15]. With a sample size of 40 per group, an alpha of 0.05 and an intraclass correlation of 0.6 (between pre and post repeated measurements) we would have 85–99% power to detect a 3–10 point difference in IDS-SR score between each of our experimental groups compared to the control group [45]. In the previous RCT, about 10% of the enrolled participants withdrew prematurely or only completed the ESM study partially [15]. Given that our study has less face-to-face contact, commitment to the study protocol might be lower in our sample. To ensure we will have 120 participants who completed the study randomized over three conditions, we will enroll 150 participants in the study (i.e., 50 per study arm).

#### Within-person analyses

Participants allocated to one of the two ESM modules will record data using their smartphone five times a day for 28 days in their natural environment (*N* = 140 measurements). This will provide ample data for the feedback reports, which will mainly comprise descriptive statistics and graphs (e.g., on daily fluctuations in mood and week-by-week changes in average PA and NA). VAR modeling will be used to provide participants feedback on temporal relationships between variables (e.g., PA and physical activity). Exact sample size calculations are not possible in studies using VAR, as it is typically unclear in such studies what effect size can be expected. This is because analyses are personalized and the direction of causality and the number of lagged influences in the system under investigation are usually unknown [29]. However, previous work from our and other groups suggests that a number of 60–90 measurements is sufficient to reliably identify reciprocal associations between variables (e.g., [12, 13, 46]).

#### Qualitative analyses

Qualitative data will be collected through in-depth individual interviews. The aim of the interviews is to provide viewpoints on the added value of the ESM-modules based on participants′ personal experiences. Data collection will be continued until no new themes emerge from the data (data saturation procedure); for our confined research question the estimated sample size is ~ 8–10 participants per module [47].

### Statistical analyses

#### Efficacy of the ESM-modules

The data have a hierarchical structure, because multiple IDS-SR assessments will be clustered within participants. Therefore, multilevel regression analyses will be used with the two-way interaction between time (in weeks) and treatment allocation as fixed effects, participants as random intercept and a (linear or quadratic) random slope for time (similar to [15]). This way, we can examine the effect of time on depressive symptoms (across 6 IDS-SR measurements), the effect of group (3 levels: Do-module, Think-module, Control), and the interaction between time and group separately. Similar analyses will be performed to investigate the impact of the ESM-modules on empowerment (NEL) and functioning (OQ-45). In order to take into account individual differences regarding the time interval between the start date of the participation in ZELF-i and the start date of the treatment at the mental health organization, a covariate “start date treatment – start date ZELF-i” will be included in the analyses. Thresholds for statistical significance will be set at *p* < .05.

#### Cost-effectiveness of the ESM-modules

Self-report instruments were completed assessing costs (TIC-P), depressive symptoms (IDS-SR) and quality of life (Euroqol-5D). First, a cost-effectiveness analysis will be performed with the IDS-SR as primary outcome measure. That is, cost effectiveness will be expressed in terms of a ratio where the denominator is a gain in health operationalized as a decrease in depressive symptomatology and the numerator is the cost associated with the health gain (i.e., direct (medical) costs and indirect (productivity losses) costs) assessed during the ESM and follow-up period. The incremental cost-effectiveness ratio will be determined on the basis of incremental costs and effects of ZELF-i (Do-module and Think-module) compared to usual care. Second, a costs-utility analysis will be performed with quality adjusted life years as the primary outcome measure (based on the EuroQol-5D-3 L, a generic, self-report instrument).

#### Patient-experienced utility of the ESM-modules

The patient-experienced utility of the ESM-modules will be analysed in two ways: quantitatively in all participants in the ESM arms through an evaluation questionnaire, and qualitatively through in-depth semi-structured interviews in a subsample. Quantitative data will be presented using descriptive statistics (mean, standard deviation). Two sample t-tests (or a non-parametric variant) will be used to examine differences between participants in the Do-module and Think-module. Qualitative research produces textual data in the form of transcripts and observational field notes, which will be explored inductively using content analysis to generate categories and explanations [25]. For the interviews, a topic list has been created by the researchers. After 4 interviews (2 Do-module, 2 Think-module), this topic list will be reviewed and newly collected issues will be incorporated according to interim analysis [25]. The adjusted topic list will be used for data collection in the successive interviews [48]. This has the advantage that the researchers can refine their questions and stay open to new relevant themes and experiences, which could be in contradiction to previously reported experiences [25]. Data collection and data analysis will be continued until no new themes emerge from the data (i.e., data saturation has been reached).

### Missing data

Participants who drop-out during the intervention phase will be asked to fill out the follow-up outcome questionnaires. Participants who complete less than 75% of the ESM measurements during the intervention period, will receive descriptive feedback in their final report but not feedback based on statistical models. Analyses will be performed to examine whether the attrition rate relates to participants’ demographic characteristics and levels of depression. Missing data will be imputed by means of multiple imputation.

## Discussion

Recent studies have suggested that ESM may be an effective therapeutic tool for patients with depression [11], which calls for further research in regular outpatient care. The present pragmatic RCT will be the first to examine the efficacy of self-monitoring and personalized feedback as a stepping stone for the treatment of depressive complaints in terms of added impact on depressive symptomatology, empowerment and social functioning. By starting directly after intake with ESM, we hope that patients can make the most out of common wait list periods, and commence subsequent treatment programs with a kick-start. ZELF-i is also the first study to evaluate two different ESM intervention modules targeted at both of depression’s core symptoms (ESM “Do-module” and ESM “Think-module”), which will harbor knowledge about what type of ESM-derived personalized feedback is most effective. Lastly, it is the first study that uses a combination of qualitative and quantitative methods to evaluate the patient-experienced utility of ESM with personalized feedback as an intervention for depression. Thus, the proposed study could provide more definitive answers regarding the added value of ESM as a therapeutic tool for depression and potentially improve psychiatric care for depression.

There are a couple of methodological issues that should be considered beforehand. First, as participants need to be motivated to fill out 5 measurements per day for 28 days, the findings may not be generalizable to all depressed patients. In a comparable ESM study, 39% of the invited patients declined to participate [15]. That said, generalizability issues play a role in virtually all intervention studies, and this response rate is comparable to other RCTs in patients with depression (e.g., [49]). Once enrolled, 86% of the depressed participants completed the 6-weeks intervention period in the above-mentioned study [15], indicating that ESM was feasible and acceptable for the majority of depressed patients. Second, the time between the ZELF-i intervention and the start of the regular treatment will not be the same across participants, because waiting list periods vary across time and treatment locations. We will examine the impact of this variation in our analyses. Third, there could be differences between the participating mental health care institutions in the degree to which ZELF-i is accepted by the practitioners and integrated in care as usual. Therefore, we will explore potential differences in the added value of ZELF-i between the different locations. Lastly, we cannot exclude there will be a potential beneficial effect of the extra attention that participants in the intervention groups receive compared to the control group. However, we deemed a sham condition in which participants fill out irrelevant questionnaires too demanding for patients. Furthermore, the extra face-to-face contact is minimal compared to previous research [15]; participants in the intervention groups only have one extra appointment with a research assistant (debriefing) compared to the control group.

## Additional files

Additional file 1: **Table S1.** Diary items. (DOCX 25 kb)

Additional file 2: **Table S2.** Overview of instruments. (DOCX 15 kb)

## Abbreviations

DSM-IV: Diagnostic statistical manual of mental disorders, fourth edition
ESM: Experience sampling method
IDS-SR: Inventory of depressive symptomatology - self-report
NA: Negative affect
NEL: Dutch empowerment questionnaire
OQ-45: Outcome questionnaire-45
PA: Positive affect
RCT: Randomized controlled trial
VAR: Vector autoregression
