# Supplementary material for: Self-monitoring and personalized feedback based on the experiencing sampling method as a tool to boost depression treatment: a protocol of a pragmatic randomized controlled trial (ZELF-i)
Source: BMC Psychiatry. 2018 Sep 3;18:276. doi: 10.1186/s12888-018-1847-z (PMC6122175; doi:10.1186/s12888-018-1847-z)
Supplement: Supplementary file 1 — Table S1. Diary items. (DOCX 25 kb) [file 12888_2018_1847_MOESM1_ESM.docx]

Table S1: Diary items

| Module | Dutch | Translation | Response labels | Range | Description |
| --- | --- | --- | --- | --- | --- |
| Both | Hoe gaat het op dit moment met u? | How are you feeling at this moment? | "Very bad" to "Very good" | 0-100 (slider) | Current well-being |
| Both | Is dit de eerste meting die u invult na de nacht? | Is this the first measurement that you are filling out after the night? | Yes/No | 0-1 | Verification question: if no, skip the next question |
| Both | Hoe heeft u de afgelopen nacht geslapen? | How did you sleep last night? | "Very bad" to "Very good" | 0-100 | Sleep quality |
| Both | Ik voel me opgewekt | I feel cheerful | "Not at all" to "Very much" | 0-100 | Positive affect, high arousal |
| Both | Ik voel me geïrriteerd | I feel irritated | "Not at all" to "Very much" | 0-100 | Negative affect, high arousal |
| Both | Ik voel me somber | I feel down | "Not at all" to "Very much" | 0-100 | Negative affect, low arousal |
| Both | Ik voel me ontspannen | I feel relaxed | "Not at all" to "Very much" | 0-100 | Positive affect, low arousal |
| Both | Ik voel me tevreden | I feel satisfied | "Not at all" to "Very much" | 0-100 | Positive affect, low arousal |
| Both | Ik voel me onverschillig | I feel indifferent | "Not at all" to "Very much" | 0-100 | Negative affect, low arousal |
| Both | Ik voel me energiek | I feel energetic | "Not at all" to "Very much" | 0-100 | Positive affect, high arousal |
| Both | Ik voel me angstig | I feel anxious | "Not at all" to "Very much" | 0-100 | Negative affect, high arousal |
| Both | Ik voel me enthousiast | I feel enthusiastic | "Not at all" to "Very much" | 0-100 | Positive affect, high arousal |
| Both | Ik voel me kalm | I feel calm | "Not at all" to "Very much" | 0-100 | Positive affect, low arousal |
| Both | Ik voel me lusteloos | I feel listless | "Not at all" to "Very much" | 0-100 | Negative affect, low arousal |
| Both | Ik voel me gestrest | I feel stressed | "Not at all" to "Very much" | 0-100 | Negative affect, high arousal |
| Think | Mijn hoofd zit vol gedachten | My head is full of thoughts | "Not at all" to "Very much" | 0-100 | Mindfulness |
| Both | Ik ben moe | I am tired | "Not at all" to "Very much" | 0-100 | Energy level |
| Both | Ik heb honger | I am hungry | "Not at all" to "Very much" | 0-100 | Need for food |
| Both | Ik ervaar lichamelijk ongemak | I experience physical discomfort | "Not at all" to "Very much" | 0-100 | Physical discomfort |
| Both | Ik ben onder de invloed van alcohol en/of drugs | I am under the influence of alcohol and/or drugs | "Not at all" to "Very much" | 0-100 | Impact of alcohol/drugs |
| Do | Ik vond de afgelopen drie uur leuk | I enjoyed the previous three hours | "Not at all" to "Very much" | 0-100 | Experienced pleasure |
| Do | Ik heb gedaan wat ik wilde/moest doen | I have done what I wanted/had to do | "Not at all" to "Very much" | 0-100 | Motivation (execution) |
| Do | Ik ben lichamelijk actief geweest | I have been physically active | "Not at all" to "Very much" | 0-100 | Physical activity |
| Do | Ik had het druk | I have been busy | "Not at all" to "Very much" | 0-100 | Business |
| Do | Ik ben thuis geweest | I have been at home | "Not" to "All the time" | 0-100 | Time spent at home |
| Do | Ik ben in prettig gezelschap geweest | I have been in pleasant company | "Not" to "All the time" | 0-100 | Time spent in pleasant social context |
| Do | Ik ben in de buitenlucht geweest | I have been in the outdoor air | "Not" to "All the time" | 0-100 | Time spent outside |
| Do | Welke activiteiten heeft u de afgelopen drie uur gedaan? | What activities did you do in the past three hours? | Tick the box if "yes" | 0-13 | Activities in the past 3 hours (multiple answers possible) |
|  | (1) Werken | (1) Working |  |  |  |
|  | (2) Huishouden | (2) Household chores |  |  |  |
|  | (3) Studeren | (3) Studying |  |  |  |
|  | (4) Zelfverzorging | (4) Self-care |  |  |  |
|  | (5) Onderweg zijn | (5) Commuting |  |  |  |
|  | (6) Zelf iets maken | (6) Creating something |  |  |  |
|  | (7) Er zelf op uit gaan | (7) Going out on my own |  |  |  |
|  | (8) Met anderen iets ondernemen: uit | (8) Doing something with others: out of the house |  |  |  |
|  | (9) Met anderen iets ondernemen: thuis | (9) Doing something with others: at home |  |  |  |
|  | (10) Rusten | (10) Resting |  |  |  |
|  | (11) Lezen/kijken/luisteren | (11) Reading/watching/listening |  |  |  |
|  | (12) Indirect contact met anderen | (12) Indirect contact with others |  |  |  |
|  | (13) Iets anders | (13) Something else |  |  |  |
|  | (14) Ik heb niets gedaan | (14) I did not do anything |  |  | NB If "Yes" all other activities are ticked off |
| Think | Ik heb nagedacht over hoe ik mij voelde | I thought about how I was feeling | Not at all to "Very much" | 0-100 | Focus on feelings |
| Think | Ik heb gepiekerd | I have been brooding over things | Not at all to "Very much" | 0-100 | Brooding |
| Think | Welke negatieve gebeurtenissen hebben er de afgelopen drie uur plaatsgevonden? | What negative events took place in the past three hours? | Tick the box if "yes" | 0-10 | Negative events in the past 3 hours (multiple answers possible) |
|  | (1) Ik had vervelend contact met anderen | (1) I had unpleasant contact with others |  |  |  |
|  | (2) Ik werd buitengesloten of genegeerd | (2) I was excluded or ignored |  |  |  |
|  | (3) Ik had last van het leed van een ander | (3) Another person's suffering caused me sorrow |  |  |  |
|  | (4) Ik zat onder (tijds) druk | (4) I was under (time) pressure |  |  |  |
|  | (5) Ik heb slecht gepresteerd | (5) I performed poorly |  |  |  |
|  | (6) Ik heb negatieve feedback gekregen | (6) I received negative feedback |  |  |  |
|  | (7) Ik heb fysiek ongemak geleden | (7) I experienced physical discomfort |  |  |  |
|  | (8) Ik had een tegenvaller | (8) I had a setback |  |  |  |
|  | (9) Ik heb een saaie of vervelende activiteit gedaan | (9) I did a boring or annoying activity |  |  |  |
|  | (10) Er was (nog) een andere vervelende gebeurtenis | (10) There was an other unpleasant event |  |  |  |
|  | (11) Er waren helemaal geen vervelende gebeurtenissen | (11) There were no unpleasant events at all |  |  | NB If "Yes" all other activities are ticked off |
| Think | Hoe onplezierig waren de negatieve gebeurtenissen bij elkaar? | How unpleasant were the negative events together? | "Not at all" to "Very much" | 0-100 | Overall valence of negative events |
| Think | Ik heb negatieve gedachten gehad over mezelf/mijn situatie | I had negative thoughts about myself/my situation | "Not at all" to "Very much" | 0-100 | Negative thoughts |
| Think | Welke positieve gebeurtenissen hebben er de afgelopen drie uur plaatsgevonden? | What positive events took place in the past three hours? | Tick the box if "yes" | 0-10 | Positive events in the past 3 hours (multiple answers possible) |
|  | (1) Ik heb gelachen of leuk gecommuniceerd met iemand | (1) I laughed or had fun communication with others |  |  |  |
|  | (2) Ik voelde me onderdeel van een groep | (2) I felt part of a group |  |  |  |
|  | (3) Ik genoot van de vreugde van een ander | (3) Another person's happiness caused me joy |  |  |  |
|  | (4) Ik heb iets voor iemand anders gedaan waar diegene blij mee was | (4) I did something for another person they were happy with |  |  |  |
|  | (5) Ik heb goed gepresteerd | (5) I performed well |  |  |  |
|  | (6) Ik heb positieve feedback gekregen | (6) I received positive feedback |  |  |  |
|  | (7) Ik heb lichamelijk genot beleefd | (7) I experienced physical pleasure |  |  |  |
|  | (8) Ik had een meevaller | (8) I had a lucky break |  |  |  |
|  | (9) Ik heb een leuke activiteit gedaan | (9) I did a fun activity |  |  |  |
|  | (10) Er was nog een (andere) plezierige gebeurtenis | (10) There was an other pleasant event |  |  |  |
|  | (11) Er waren helemaal geen plezierige gebeurtenissen | (11) There were no pleasant events at all |  |  | NB If "Yes" all other activities are ticked off |
| Think | Hoe plezierig waren de positieve gebeurtenissen bij elkaar? | How pleasant were the positive events together? | "Not at all" to "Very much" | 0-100 | Overall valence of positive events |
| Think | Ik heb positieve gedachten gehad over mezelf/mijn situatie | I had positive thoughts about myself my situation | "Not at all" to "Very much" | 0-100 | Positive thoughts |
| Both | Is dit de laatste meting van de dag? | I this the last measurement of the day? | Yes/No | 0-1 | Verification question: if yes, the phrasing of the next 3 questions changes and 4 questions are added |
| Do | Ik heb zin in de komende drie uur/morgen | I am looking forward to the next three hours/tomorrow | "Not at all" to "Very much" | 0-100 | Anticipatory pleasure |
| Do | Ik neem me voor om straks/morgen bepaalde dingen te gaan doen | I intend to undertake certain things in the next three hours/tomorrow | "Not at all" to "Very much" | 0-100 | Motivation (intention) |
| Think | Ik maak me zorgen over wat er de komende drie uur/morgen gaat gebeuren | I am worried about what will happen in the next three hours/tomorrow | "Not at all" to "Very much" | 0-100 | Worrying |
| Both | Ik kon aan wat op mijn pad kwam | I was able to handle today's challenges | "Not at all" to "Very much" | 0-100 | Coping |
| Both | Ik kon mij ertoe zetten dingen te doen die ik wilde/moest doen | I could motivate myself to do the things I wanted/needed to do | "Not at all" to "Very much" | 0-100 | Motivation (execution) |
| Both | Ik kon negatieve gedachten makkelijk loslaten | I could easily let go of negative thoughts | "Not at all" to "Very much" | 0-100 | Mindfulness |
| Both | Vandaag ging het met mij… | Today I felt… | "Very bad" to "Very good" | 0-100 | Retrospective well-being |
| Both | Deze meting stoorde mij | This measurement bothered me | "Not at all" to "Very much" | 0-100 | Hinderance by measurement |
