# Supplementary material for: Self-monitoring and personalized feedback based on the experiencing sampling method as a tool to boost depression treatment: a protocol of a pragmatic randomized controlled trial (ZELF-i)
Source: BMC Psychiatry. 2018 Sep 3;18:276. doi: 10.1186/s12888-018-1847-z (PMC6122175; doi:10.1186/s12888-018-1847-z)
Supplement: Supplementary file 2 — Table S2. Overview of instruments. (DOCX 15 kb) [file 12888_2018_1847_MOESM2_ESM.docx]

**Table S2. Overview of instruments**

| **Instrument** | **Purpose** |  | **Baseline**  **(pre-ESM)** | **Post-ESM** | **1 Month FU** | **2 Month FU** | **3 Month FU** | **6 Month FU** |
| --- | --- | --- | --- | --- | --- | --- | --- | --- |
|  |  |  | **T0** | **T1** | **T2** | **T3** | **T4** | **T5** |
| ***Outcomes*** | | | | | | | | |
| IDS-SR | Depressive Symptoms |  | X | X | X | X | X | X |
| OQ-45 | Functioning |  | X | X | X | X | X | X |
| NEL | Empowerment |  | X | X | X | X | X | X |
|  | | | | | | | | |
| ***Costs-effectiveness*** | | | | | | | | |
| TiC-P | Illness costs |  | X | X* |  |  | X | X |
| Euroqol-5D | Quality of life |  | X | X |  |  | X | X |
|  |  |  |  |  |  |  |  |  |
| ***Patient-experienced utility*** | | | | | | | | |
|  | ESM Evaluation |  |  | X |  |  |  |  |
|  | Interview* |  |  |  |  | (X) |  |  |
|  |  |  |  |  |  |  |  |  |
| ***Baseline characteristics*** | | | | | | | | |
|  | Demographics |  | X |  |  |  |  |  |
| LEIDS-R | Cognitive Reactivity |  | X |  |  |  |  |  |
| TAS-20 | Alexithymia |  | X |  |  |  |  |  |

*Note.* T = measurement wave, FU = follow-up (post-ESM),

* These questionnaires normally assess costs across a 3-month period. Post-ESM, the time window will be restricted to the 28-day intervention period only. ** The interview is optional and will take place before the 2 month follow-up.
